# Developmental Origins of Cardiovascular Disease: Understanding High Mortality Rates in the American South

**DOI:** 10.3390/ijerph182413192

**Published:** 2021-12-14

**Authors:** Garrett T. Senney, Richard H. Steckel

**Affiliations:** 1 Office of the Comptroller of the Currency, 400 7th Street SW, Washington, DC 20219, USA; 2 Economics, Anthropology, and History Departments, Emeritus, Ohio State University 1945 N. High St., Columbus, OH 43210, USA; Steckel.1@osu.edu

**Keywords:** developmental origins, cardiovascular disease, health, I15, N32, O15

## Abstract

While many social scientists view heart disease as the outcome of current conditions, this cannot fully explain the significant geographic disparities in cardiovascular disease (CVD) mortality rates in the USA. The developmental origins hypothesis proposes that CVD vulnerability is created by poor conditions in utero that underbuilds major organs relative to those needed to process lush nutrition later in life. The American South underwent an economic transformation from persistent poverty to rapid economic growth in the post-World War II era. We use state-level data on income growth and current conditions to explain variation in CVD mortality rates in 2010–2011. Our proxy for unbalanced physical growth, the ratio of median household income in 1980 to that in 1950, has a large systematic influence on CVD mortality, an impact that increases dramatically with age. The income ratio combined with smoking, obesity, healthcare access, and education explain more than 70% of the variance in CVD mortality rates.

## 1. Introduction

**Figure 1:**
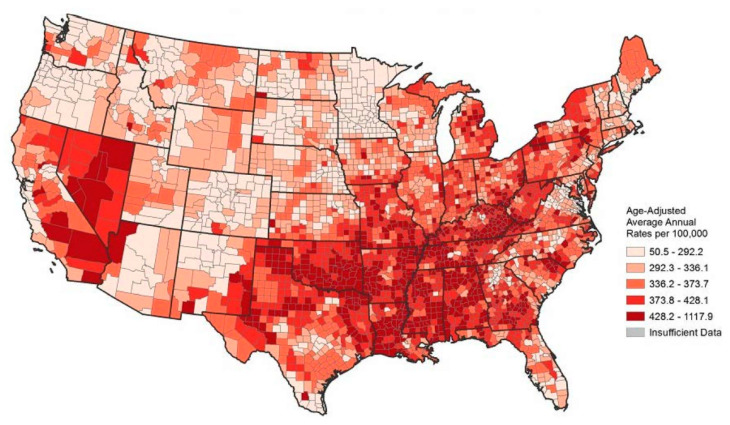
Age-Adjusted County Level CVD Mortality Rate in 2011-13. Source: CDC/NCHS, National Vital Statistic System.

Cardiovascular disease, also known as CVD, is a costly health problem that absorbs nearly $450 billion annually in health care expenditures [1]. Nearly one-quarter of all deaths in the U.S. stemmed from heart disease in 2010 [2]. Since individuals with ongoing cardiovascular conditions (e.g., coronary heart disease) are more than twice as likely to have a stroke than those who do not, one could plausibly add stroke deaths, which would make the total approximately 30% [3]. However, this health burden is not spread uniformly across the country. Researchers recognize that the South has long had poor health outcomes [4,5]. One can see from Table 1 that eight of the top ten states with the highest average age-adjusted, all causes mortality rate from 1999 to 2010 are located in the South, a result that also holds for the nation’s leading cause of death, CVD, as defined by the death registration system. Figure 1 illustrates this disparate regional pattern in CVD mortality quite strikingly.

Some argue the American South’s poor showing follows from concurrent conditions, such as low levels of education, lack of exercise, a poor diet, and lack of healthcare access. While this idea has merit, past research on diabetes and hypertension indicates that the developmental origins hypothesis can contribute to understanding the current CVD mortality disparity. The developmental origins hypothesis is rooted in the fact that intergenerational transitions from persistent poverty to prosperity promotes chronic non-infectious diseases in adults. Poor nutrition and stress in utero compromise the construction of vital organs and degrade processes that regulate physiological systems in later life, making these individuals vulnerable to chronic diseases as adults [6,7,8,9].

Rapidly improving socioeconomic conditions that follow persistent poverty creates non-harmonious growth, whereby vital organs are prepared in utero for the individual to live a lean life but face a lush one as an adult, which elevates the risk of CVD complications. For example, a representative study of Puerto Ricans aged 60–74 found that after controlling for standard risk factors the probability of heart disease was 65% higher among individuals who were born during seasons in which the incidence of disease and poor nutrition were higher [10]. Researchers have even found some suggestive evidence that experiences of deprivation in one generation can be transmitted not only to immediate offspring but to further generations as well [11,12].

We evaluate the hypothesis that rapidly improving socioeconomic conditions preceded by intergenerational poverty causes a higher likelihood of CVD. The basis for the hypothesis is found in earlier epidemiological studies [7]. Families who lived in poverty for generations are more likely to have children with organs optimized for survival in a life of deprivation. Under the developmental origins hypothesis, if these families suddenly experienced intergenerational prosperity and nutritional abundance, then these children would have greater propensity to have CVD as adults.

In this paper, we argue that the geographic disparity in CVD can be partial explained by the rapid and uneven economic growth that began in the 1950s. Finding a suitable socioeconomic proxy for non-harmonious growth is a challenge for this line of research. The economic history of the South, however, creates a fortunate opportunity for study. The American South was poor for decades relative to the national average following the Civil War but grew faster than the national average beginning in the 1960s [13]. Our approach follows the efforts of [14,15], who show that the states with higher diabetes and hypertension prevalence in 2010 correlate well with the states that had the greatest median per capita income growth between 1950 and 1980. If the developmental origins hypothesis has merit, then the ratio of median household income in 1980 to that in 1950 should be a useful proxy for non-harmonious growth for CVD as well.

**Figure 2:**
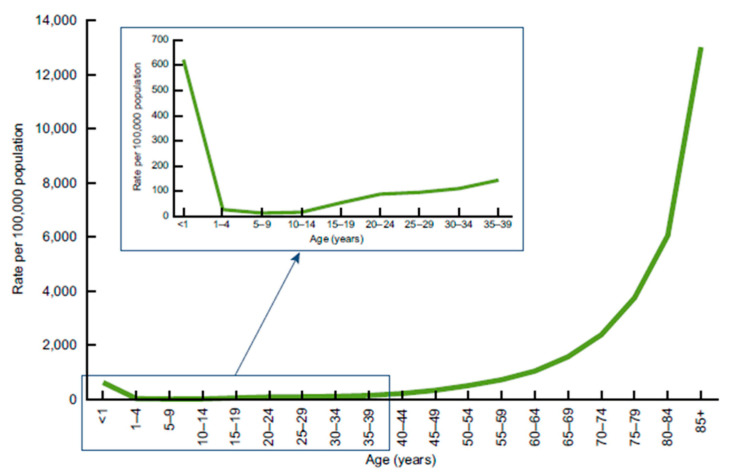
Age-Specific CVD Mortality rates in 2009. Source: CDC/NCHS, National Vital Statistics System.

The data in Figure 2 helps us organize our approach to CVD mortality. Age differences in CVD mortality are large while gender and racial differences are rather small [2]. The gradient of CVD mortality rates rises sharply beyond middle age, increasing from 12.3% at ages 25–44 to 26.5% at ages 65+. Therefore, we focus on mortality patterns of whites by age, but in future research we plan to investigate racial differences in CVD mortality rates. Using current conditions commonly used by social scientists in the analysis of CVD mortality (e.g., obesity, education) along with income growth, we are able to explain over 70% of the variance in CVD mortality rates across states in the USA.

## 2. Possible Mechanisms

What distinctive features of the South might have translated rapid income growth into adolescent weight gain, and eventually elevated CVD mortality rates? Higher incomes alone enabled families to purchase more food, an item that would have been high on the list of priorities for southern families, which were especially poor. This relationship is enshrined in Engle’s Law, the poorer the family the greater the outlay of income on food. The proportion of income spent on food is a good measure of the standard of living of a population, and numerous modern studies substantiate this conclusion [16].

Economic historians know that rapid economic change creates many new opportunities but also disrupts family life, as studies of industrialization make clear [17,18]. As southern agriculture mechanized and food became cheaper, farm women joined the labor force, often taking jobs in food processing plants, the service sector, and government installations [19]. To realize these opportunities, families may have relocated and members may have acquired new skills and adopted new commuting patterns, all of which were stressful. Some people find that food allows them to cope with stress, and they eat more and gain weight [20].

Southern mothers were overwhelmingly responsible for domestic chores, like preparing meals, which did not change when they started to enter the labor force. However, they now had less time for home production and less opportunity to supervise the eating habits of their children. They may have used their earnings to purchase more of the newly available processed food, which often has lower nutritional quality [21]. Another outcome is that children, who once had to work manual jobs to help support the family, were released from labor by their parents’ higher incomes, adding to net nutrition and thereby contributing to weight gain [22]. Furthermore, families that had both parents employed outside the home had less opportunity to supervise the eating and free time of their children. These children, who’s after school time was once occupied by field labor or chores, were now free to enjoy more leisure. Ownership rates of TV increased over 30-fold for Southerners from 1950 to 1970 (Census 1950 and 1970).

The possible role of southern culture and the interaction of diet and traditional attitudes towards physical exercise is another factor. Unlike other regions, agriculture was the dominant employer in the South prior to the beginning of industrialization after the middle of the 20th century. Relative to other regions, southern farmers were slow to adopt the tractor, and mules lingered on small farms until well into the 1950s [23]. Mechanization of the harvest was difficult to accomplish in the region’s most important crops, cotton and tobacco, and relief from field labor came late relative to other regions [24]. Mechanical cotton pickers largely replaced hand labor between the late 1940s and the 1960s, but hand methods persisted on small farms for a decade or more [25,26].

Southern customs were fashioned by a long history of physical labor in the fields that welcomed rest at the end of the day. The South was not a region where habits of recreational exercise and health club memberships readily replaced a decline in caloric expenditure associated with a reduction in physical labor. In 2007, the share of the population belonging to health clubs ranged from a low of 6.3% in West Virginia to a high of 21.8% in Colorado [27]. There is a strong negative correlation between CVD morality and health club membership. In every state in the high CVD risk region of the South, the share of the population belonging to a health club is below the national average of 15.5%.

Rising incomes enabled families to replace walking with a less taxing form of transportation and hauling, the automobile. According to the Federal Highway Administration’s National Household Travel Survey, from 1950 to 1980, the number of miles traveled in a personal vehicle per capita increased by more than 2.3 times while the number of vehicle registration per capita increased by about 2.5 times for the South. That was by far the biggest increase of any region in the country.

The Southern food ways had deep roots in the 19th century when pioneer farmers planted corn and created swine herds [28]. For most of the year the hogs foraged and then early in the fall farmers assembled them for fattening on corn. Meat processing occurred after the first cold spell. Fat was rendered into lard and the hams and shoulders were salted, smoked, and stored. As long as pork was available, these farmers ate it daily, accompanied by various forms of corn processed into bread, grits, or hominy. According to USDA Nationwide Food Surveys from 1955, 1965, and 1977, southerners on average consumed 4% more meat, 5% more fats and oils, and 18% more sugar and sweets (by weight) weekly than the national average.

When available, vegetables were usually fried or boiled with a piece of lard or pork. Sweet potatoes were also common fare in the diet because they required minimal cultivation and they could be stored for months in underground cellars. According to USDA Nationwide Food Surveys from 1955 and 1977, southerners consumed about 12% fewer fresh fruits by weight than the national average. This lower consumption pattern still holds today, where southern states have the highest proportions of adults who self-report consuming one or less fruit and one or less vegetable in any form per day (http://www.cdc.gov/nutrition/downloads/State-Indicator-Report-Fruits-Vegetables-2013.pdf accessed on 12 June 2019). By the 20th century the price of wheat began to decline, and new methods of milling and distribution enabled even poor southern farmers to buy flour in bulk. Biscuits became a staple of the diet. In the environment of income growth, the declining food prices, the reduction of work, and changing roles within the family created the perfect storm to make a generation of Southerners especially vulnerable to chronic adult diseases.

## 3. Data

We use detailed data of the underlying cause of death from the CDC’s Wide-ranging Online Data for Epidemiologic Research (WONDER) to examine the impact median household income changes have on the white CVD mortality rate in 2010–2011. The mortality rate is measured at the state-level and includes deaths which had a recorded underlying cause within the ICD I00I99 categories; diseases of the circulatory system which includes disorders like hypertensive diseases, cerebrovascular diseases, and pulmonary heart disease. The CDC collects this data from official death certification published by the county governments. We examine crude death rates per 100,000 people in 2010 and 2011 for individuals in the age groups of 55–64, 65–74, 75–84, and 55–84. The individuals in these age cohorts were born between 1926 and 1955.

Researchers have identified numerous factors associated with CVD, including lifestyle, family history, and genetics. Genetic and family-history research in the area has mushroomed in the past decade, and while researchers can point to progress, the information acquired explains relatively little of the variation in deaths across individuals and populations [29,30,31,32]. Lifestyle conditions have been the most intensively studied factors such that a standard profile of risks has emerged, encompassing obesity, smoking, physical inactivity, a high-fat diet, and psychological stress [33,34,35]. Also implicated are uncontrolled hypertension and diabetes as well as low education and poor access to medical care. It is also worth noting, however, that one-half of individuals with CVD lack any of the conventional risk factors [36], and therefore much remains to be explained concerning the origins of this disease.

As such, we use state level data on average levels of obesity, smoking, and educational attainment from the CDC’s Behavioral Risk Factor Surveillance System (BRFSS) in 2010. The BRFSS is a cross-sectional telephone survey conducted by state health departments with technical assistance from the CDC. The respondents are asked to self-report basic demographics (race, gender, height, and weight) as well other health related conditions. Smoking was indicated by a dummy (0,1) variable indicating whether the individual smoked more than 10 cigarettes per day. The BRFSS staff then calculate each respondent’s body mass index, which is coded as obese if the BMI is greater than or equal to 30. Respondents also report their highest grade or year of school completed. An indicator variable was created to for individuals whose highest level education was high school.

Median household income is taken directly from the U.S. Census of Population for 1950 and 1980. The nominal income was adjusted using the Federal Reserve Bank of Minneapolis’ Chained-weighted CPI with 1982–1984 as the base years. The growth rate is found by dividing median household income in 1980 by median household income in 1950. We also use information on the number of active physicians per capita in 2011 from State Physician Workforce Data Report put out by the Association of American Medical Colleges. Table 2 and Table 3 provide summary statistics of the variables used in this analysis.

We recognize that interstate migration can distort the empirical analysis, for which we do not have a fully satisfactory solution. We contend with the problem by using weighted regressions employing different measures of population turnover within the state. The turnover measure is based on domestic in- and out-migration data coming from the U.S. Census. We define turnover as the sum of immigration and out-migration for a state, which is used to measure chronological change in the state population. A larger turnover value implies the actual composition of individuals residing in the state have changed dramatically over time, which could potentially be masked by simply using net migration numbers. For example, if the entire state population departed, but was replaced by the exact same number of people arriving, the net migration for the state would be zero. We use a state’s average turnover from 1950 to 1980 as regression weight, thereby giving less weight to states that had high turn-over.

The average turnover from 1950 to 1980 does not recognize the origins of the new residents which is does not account for the fact intra-region migration is less problematic for our analysis than inter-region. Therefore, we also collect data from the U.S. Census on the proportion of each state’s adult residents in 2010 who were born in the South. While this measure does not capture more general migration, it better represents the mixture of individuals with high probabilities of arriving from high growth income areas (i.e., the South) who were vulnerable to CVD. As a robustness check, we also estimate our model using the share of the population born in the South as a weight.

## 4. Methods

The OLS model we estimate is:

$$MR_{i}=\beta_{0}+\beta_{1}HI_{i}+\theta X$$

where $MR_{i}$ is the CVD mortality rate of whites in state *i* in 2010–2011, $HI_{i}$ is the ratio of median household income of whites in 1980 to that in 1950, and *X* is a set of covariates that control for other conditions. Risk factors for CVD mortality include low levels of education, active physicians per capita, smoking, and obesity [37,38]. These variables are measurable at the state level and are included as the controls in estimating the effect of income change. Under the proposed hypothesis, the coefficient *β*_1_ should be positive, large, and statistically significant.

Population turnover is relevant because current mortality rates are hypothesized to be a function of conditions that existed from 1950 to 1980. It would be ideal if there was no population turnover from 1950 to 2010, such that the population under study was constant, or at least undisturbed by people moving in or out of the state. Of course, that is not true, and the amount of turnover varies across states and must be considered in using state-level data. The issues at hand are how to incorporate turnover into the analysis, and whether there was enough stability in state populations to yield a systematic relationship between past conditions and current outcomes. If migration heavily contaminated the relationship and the error term was large, one would expect to find a low R^2^ and coefficients that were statistically insignificant, or worse, a statistical outcome that contradicted well-founded results, such as smoking or obesity being beneficial for CVD. Plausible outcomes for well-researched variables would lend credence to the measured impact of past income change on mortality rates. We use a weighting scheme that reduces the importance of high-turnover states in the analysis by weighting states by their inverse of turn-over rates.

## 5. Results

**Figure 3:**
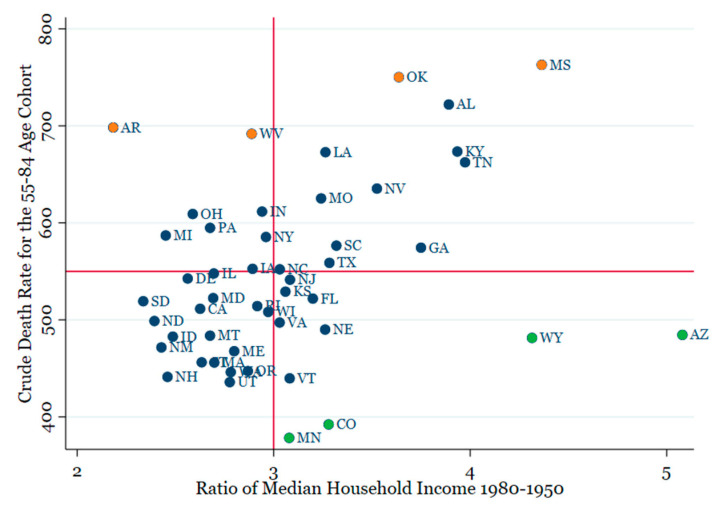
Scatter plot of Median Household Income vs. Death Rate for the 55–84 Age Cohort.

Figure 3 presents a scatter diagram of the statistically significant relationship between the mortality rate at ages 55–84 on the income ratio. A vertical line at 3 and a horizontal line at 550 are added for reference. The scatter diagrams are similar using the other dependent variables. As expected, most of the states on the top right-hand side of the graph (largest median income growth and highest death rates) are located in the South. Several interesting outliers suggest that an expansion of the model would be useful. In particular, the four largest outliers above the regression line (Orange dots) and the four largest below the line (Green dots) all have above/below average characteristics linked to CVD in earlier studies.

The four positive outliers have above average values of smoking, obesity, and years of education at high school or less, while all those below the line have below average values of these variables. Notably, Arkansas is third lowest and West Virginia is the lowest among all states on the scale of education (57.1% and 64.5%, respectively). Oklahoma, Louisiana, Arkansas, Alabama, and Mississippi have the second through sixth highest levels of smoking. It emerges that much of the scatter around the regression line may be explained by current conditions adversely linked to CVD.

Table 4, Table 5, Table 6 and Table 7 display the regressions with no weights, the inverse of average turnover 1950–1980, and the inverse of average turnover 1950–2000 as weights. In principle, our concerns about turnover may have been well-founded, but the empirical results are affected little by weighting. Comparing results from the non-weighted regressions, with those of the different weighted results, reveals similar patterns of coefficient sizes and statistical significance.

To further account for the effects of southern birth, Table 7 display the results of the same regression specification except using percent of the population born in the South as a weight. If the developmental origins hypothesis is correct, then southern born individuals should have been vulnerable to CVD, at least to same degree, regardless of where they lived as adults. As expected, the income variable is both statistically and economically significant in the presence of the control variables. A 50% increase in the 1950 to 1980 income growth is estimated to increase the CVD mortality rate by 10.4 per 100,000 for 55–64-year-olds and 47.9 per 100,000 for 75–84-year-olds. This is our preferred specification.

One might think that the initial level of income would affect the relationship. However, including median household income in 1950 does not qualitatively change the results, with the variable being statistically insignificant. We also test whether the relationship of the income ratio to mortality is nonlinear. We find that the coefficient of the squared term is insignificant, the other coefficients are less significant, and the adjusted R2 is lower. This suggests that the variable is irrelevant to the equation. Further, the results are essentially unaffected by using a log functional form.

The coefficient on the education control also has the expected sign. More people with less education implies that the average resident of the state is less informed about the importance of regular health maintenance, less knowledge of resources to assist in obtaining healthcare, and potentially less able to understand the medical advice received. This lack of information will generally be associated with an increase in the mortality rate [39,40]. Studies show that obesity is also a factor contributing to higher levels of mortality from CVD [41,42].

It turns out that smoking and obesity are substantially correlated (r = 0.64), and so it is difficult to obtain precise estimates of their independent effects on mortality. When entered separately each variable is statistically significant, but standard errors of the coefficients are somewhat higher when entered together. Goodness of fit tests suggest that are jointly significant and should be included together.

## 6. Robustness Check

In this section, we perform some sensitivity analysis by exploring the inclusion of other explanatory variables. The inequality within states might affect the measured income–mortality relationship to the extent that income growth generated rising inequality. This pattern called the Kuznets curve is found within many countries during industrialization [43]. The expected net effect of changes in inequality, however, is unclear. If the poor benefited relatively more from growth, then average income growth would understate their improving conditions and therefore their susceptibility to CVD. We calculated state-level Gini coefficients for income in 1980 and 1950. The ratio of the Gini coefficient 1980 to 1950 is negatively correlated with both CVD mortality and the ratio of median household income, but the relationship is rather weak and insignificant. Another measure of inequality, is the standard deviation of the income distribution. The ratio of standard deviation of income 1980 to 1950 is insignificant for all age cohorts and its inclusion doesn’t appreciably affect the other coefficients (These results are available from the authors upon request).

One of the factors behind the rapid household income growth from 1950 to 1980 is the increase in female participation in the labor force. The state with the smallest increase in the female labor force participation rate still had over 44% more women in the force labor, while the average growth is 75%. It is possible that the income growth variable is also capturing some of the effect from mothers spending more time working outside the home. To explore this factor, we add the ratio of female labor force participation rate 1980 to 1950 to the main specification. The coefficient is positive but insignificant for all age cohorts. The inclusion of this variable only slightly reduces the coefficient on the ratio of median income and the adjusted R^2^ are about the same.

Another factor to consider is the compositional shift that occurred in the sectors of employment from the 1950 to 1980. Individuals were moving away from employment in the agricultural sector to the manufacturing and service sectors. We rerun the main regression including the ratio of the share of non-agriculture employment 1980 to 1950. The coefficients are positive but insignificant. The inclusion of this variable only slightly reduces the coefficient on the ratio of median income and the adjusted R^2^ are about the same.

## 7. Discussion

It is important to recognize limitations of this statistical analysis, first in linking cohorts born near the middle of the century with economic change at the state level from 1950 to 1980. An improvement would be to link the income growth of annual birth cohorts with CVD deaths in the same birth cohorts; however, this option is unavailable with the data at hand. The approximation employed here adds some noise to the relationship, which might diminish the precision of the estimates. Nevertheless, the coefficients of interest are both statistically and economically significant, as predicted by the developmental origins hypothesis. These results add to the theoretically underpinnings of the hypothesis and provide strong credibility to the developmental origins hypothesis and its continued investigation.

Next, states are heterogeneous in all the variables employed, and so it might be desirable to use smaller geographic units of analysis such as counties that are more homogeneous. Units such as small as counties, however, often have very high rates of population turnover (above those of states) that complicate the links between income in the past with that of heart disease mortality in the present. States are not free of these problems, but they have fewer issues in this regard. Despite the shortcomings noted here, a state-level analysis explains over 70% of the variance in CVD mortality.

The coefficients on the ratio of median household income in 1980 to that in 1950 are all positive, and economically and statistically significant. Considering the three mutually exclusive groups (55–64, 65–74, 75–84), the magnitude of the effect of a rise in household income increases as the groups climb in age. The 55–64-year-old cohort would have been under 4 years old in 1950 and between 25 and 34 years old in 1980. The 75–84-year-old cohort would have been between 15 and 24 years old in 1950 and between 45 and 54 years old in 1980. This result suggests that if the income growth occurs early enough in life, while the individual is still developing, the body is better able to adjust to the changing nutritional state. The individuals who were older when the income growth occurred were less able to physiologically adapt and are the most adversely affected by the income growth [44,45].

Our analysis agrees with many studies that show smoking, obesity, lack of healthcare access, and low levels of education are all risk factors for CVD mortality. This is the case despite known problems of inference about individual behavior from regressions based on aggregate data. The ecological fallacy does not distort the interpretation of relationships measured in our results. First, we are not trying to infer individual behavior from aggregate data on smoking, obesity, and education. These relationships are already well-established at the individual level. We include these variables in the regressions to control for distortions they may impose on the income-mortality relationship. Second, examples of the fallacy contemplate distortions created by contemporaneous feed-back loops, such as immigrants who often have low levels of education, deciding where to settle based on the quality of educational opportunities. In our case, there is no such feed-back loop, or if there was it would operate with an implausibly very long lag.

We argue that income change from 1950 to 1980 affected mortality rates a half a century or more later, and it is hard to imagine how mortality rates could affect past income change. Individual death from CVD is not subject to decision in the same way as immigrants choosing a destination. A devil’s advocate might argue that older individuals with CVD chose to live in states that had high levels of past income change, but the mechanism is unclear. This might mean that CVD sufferers who lived in low-mortality states migrated to the South, perhaps for the availability of good medical care boosted by past income growth; we find that implausible and inconsistent with known migration patterns.

The coefficients on the obesity rate are positive and statistically significant for all age group cohorts. Other research has shown that carrying elevated amounts of body fat is associated with higher levels of mortality [41,42]. Furthermore, we find evidence that the obesity–mortality relationship becomes stronger with age. This result coincides with recent advancements in the medical literature [46,47].

The coefficients on smoking are positive and statistically significant for all age cohorts except for 65–74-year-olds. The overall mortality among smokers in the United States is about three times higher than that among similar people who never smoked [48]. Our results are well in line with this established literature. As a robustness check, we collected data on the percentage of people in the state who smoked at least 100 cigarettes lifetime and self-reports as currently smoking. (The data comes from the 2014 America’s Health Rankings published by the United Health Foundation, American Public Health Association, and Partnership for Prevention.) The reported regressions were all rerun using this new measure of smoking and the results are qualitatively the same. The coefficients and goodness of fit hardly change with this adjustment.

## 8. Conclusions

Our paper makes two important contributions and contributes several suggestions for additional research. First, the analysis confirms or is at least consistent with the developmental origins hypothesis as applied to CVD in explaining regional differences in mortality within the United States. These differences are large and contribute importantly to understanding sources of health inequality. Second, the impact on mortality of rapidly improving intergenerational conditions during the middle of the twentieth century increases with age across the groups 55–64 to 75–84. This suggests that the penalties of unbalanced physical growth increase when the body has less ability to adapt to a new environment.

The traditional southern diet was a disaster for heart disease when accompanied by a decline in physical labor and habits that eschewed recreational exercise. The southern diet is gradually changing but fried foods, such as chicken, catfish, and hushpuppies, remain popular to this day. Pockets of strong dietary tradition linger in many rural regions, a pattern that offers an opportunity to study CVD at the county level.

A topic untouched by the evidence analyzed here is the consequence of duration of relative poverty and affluence on CVD mortality. One might reasonably hypothesize that for a given increase in income, children of those women having had longer intergenerational experiences of poverty may have had greater susceptibility. Similarly, for a given duration of poverty, children of women having had greater increases in income would also be more susceptible. Individual-level intergenerational evidence is needed to investigate these interesting questions.

Lastly, the developmental origins hypothesis has especially relevant implications for the developing world, where vast amounts of poor families are on the verge of experiencing significant increases in relative income. Chronic adult illness, like heart disease, are projected to increase dramatically in these locations in the future [49,50].

## Figures and Tables

**Table 1:** Age-Adjusted All Cause and Heart Disease Mortality Rates per 100,000 by State.

Rank	State	All Cause	Heart Disease	Rank	State	All Cause	Heart Disease
1	West Virginia	970.2	332.2	25	Virginia	778.9	257.5
2	Kentucky	944.1	322.1	26	Illinois	777.2	273.3
3	Alabama	940.9	330.9	27	Montana	774.6	236.4
4	Mississippi	940.1	345.9	28	Maryland	766.8	266
5	Oklahoma	939.3	346.8	29	Idaho	766	247.3
6	Tennessee	909.8	316.7	30	Rhode Island	765.7	263.6
7	Arkansas	905	320	31	Washington	752.3	245.4
8	Louisiana	904.3	307.4	32	Vermont	751.6	242.2
9	Nevada	875.2	293.2	33	New Hampshire	751.1	247.2
10	Georgia	862.3	289.5	34	Nebraska	751	246.7
11	Indiana	853	295.1	35	New Jersey	749.2	267.8
12	South Carolina	849.8	275.4	36	Iowa	749	264
13	Missouri	848.6	303.6	37	Massachusetts	746.9	238.9
14	Ohio	846.7	293.7	38	Wisconsin	744.2	254
15	North Carolina	823.7	270	39	Arizona	743.3	236.6
16	Texas	810.4	279	40	Utah	741.8	228.9
17	Pennsylvania	807	283.1	41	New York	737.3	293.8
18	Wyoming	805.5	251.3	42	Colorado	735.7	222.8
19	Michigan	801.4	293.7	43	California	735.6	271.2
20	Delaware	797.8	267.4	44	Florida	729.8	243.5
21	Maine	796.6	246.1	45	South Dakota	715.4	246
22	Kansas	793.5	264.9	46	North Dakota	709.2	245.2
23	New Mexico	785.3	236.5	47	Connecticut	706.4	238.9
24	Oregon	779.7	241.3	48	Minnesota	691	204.9

Source: Centers for Disease Control and Prevention, National Center for Health Statistics. http://wonder.cdc.gov/ucd-icd10.html (accessed on 10 August 2018).

**Table 2:** Descriptive Statistics of Independent Variables.

	rMedian8050	Obesity	Smoking	≤High School	Health Club	Doc. Per Capita
Max	5.08	32.4	26.3	64.5	21.8	416
Mean	3.06	26.52	17.81	46.36	14.5	251
Median	2.95	26.75	17	45.95	14.5	243
Min	2.18	18.9	9.2	33.4	6.3	176
Std Dev	0.585	2.964	3.298	6.381	3.8	51
N	48	48	48	48	48	48

**Table 3:** Descriptive Statistics of Death Rates per 100,000 by Age Cohort.

	55–64	65–74	75–84	55–84
Max	330.1	696	1989.2	762.9
Mean	212.5	499.99	1538.1	545.94
Median	201.9	472.1	1499.5	482.05
Min	130.2	341.4	1134.9	378.3
Std Dev	51.7	91.35	193.1	92.64
N	48	48	48	48

**Table 4:** Regression results without weights.

	55–64	65–74	75–84	55–84
rMedian8050	22.26 ***	28.17 **	82.55 **	27.44 **
	(7.73)	(12.59)	(31.73)	(13.26)
≤High School	3.52 ***	7.78 ***	14.89 ***	7.94 ***
	(0.81)	(1.32)	(3.34)	(1.39)
Smoking	2.63 *	3.66 *	1.57	3.37 *
	(1.52)	(2.12)	(7.12)	(1.89)
Obesity	2.92 *	6.25 *	16.94 **	6.91 **
	(1.66)	(3.19)	(8.05)	(3.36)
Doc. per capita	−0.25 ***	−0.32 **	−0.47	−0.26 *
	(0.09)	(0.14)	(0.36)	(0.15)
Constant	−79.12	−96.98	239.40	−83.18
	(57.96)	(94.42)	(237.91)	(99.43)
N	48	48	48	48
Adj R2	0.7043	0.7486	0.6306	0.7289

Note: Standard errors in paratheses, * *p* < 0.10 ** *p* < 0.05 *** *p* < 0.01.

**Table 5:** Regression results with average turnover 1950–1980 weights.

	55–64	65–74	75–84	55–84
rMedian8050	18.64 **	26.25 *	70.92 ***	17.41 *
	(6.98)	(10.73)	(25.99)	(9.78)
≤High School	4.07 ***	8.61 ***	15.84 ***	8.64 ***
	(0.82)	(1.26)	(3.05)	(1.39)
Smoking	2.92 *	5.69 **	3.22	4.47 *
	(1.51)	(2.33)	(2.63)	(2.57)
Obesity	3.05 **	3.77 *	17.40 **	6.45 **
	(1.81)	(2.11)	(6.77)	(3.08)
Doc. per capita	−0.33 ***	−0.49 ***	−0.91 ***	−0.50 ***
	(0.08)	(0.13)	(0.31)	(0.14)
Constant	−90.87 *	−67.27	275.80	−44.74
	(52.99)	(81.49)	(197.31)	(89.78)
N	48	48	48	48
Adj R2	0.7244	0.7791	0.6993	0.7467

Note: Standard errors in paratheses, * *p* < 0.10 ** *p* < 0.05 *** *p* < 0.01.

**Table 6:** Regression results with average turnover 1950–2000 weights.

	55–64	65–74	75–84	55–84
rMedian8050	18.09 **	26.29 **	70.27 ***	16.88 *
	(6.89)	(10.53)	(25.97)	(9.43)
≤High School	4.08 ***	8.65 ***	16.05 ***	8.66 ***
	(0.80)	(1.21)	(3.01)	(1.37)
Smoking	2.86 *	5.62 **	3.40	4.53 *
	(1.49)	(2.28)	(5.62)	(2.55)
Obesity	3.26 *	3.98	17.79 **	6.66 **
	(1.79)	(2.73)	(6.73)	(3.06)
Doc. per capita	−0.31 ***	−0.46 ***	−0.85 ***	−0.48 ***
	(0.08)	(0.12)	(0.31)	(0.14)
Constant	−97.76 *	−80.20	240.80	−56.66
	(52.38)	(80.01)	(197.36)	89.71
N	48	48	48	48
Adj R2	0.7281	0.7860	0.7350	0.7751

Note: Standard errors in paratheses, * *p* < 0.10 ** *p* < 0.05 *** *p* < 0.01.

**Table 7:** Regression results with percentage of 2010 population born in the South weights.

	55–64	65–74	75–84	55–84
rMedian8050	20.79 ***	34.15 ***	95.72 ***	33.03 **
	(7.10)	(11.65)	(29.22)	(12.33)
≤High School	3.20 ***	7.32 ***	13.48 ***	6.97 ***
	(0.77)	(1.26)	(3.22)	(1.34)
Smoking	3.51 **	4.33	5.09 *	4.78 *
	(1.70)	(2.80)	(2.84)	(2.73)
Obesity	3.43 *	8.49 **	20.63 **	9.28 ***
	(1.94)	(3.19)	(8.12)	(3.38)
Doc. per capita	−0.28 ***	−0.27 *	−0.35	−0.25
	(0.09)	(0.15)	(0.37)	(0.15)
Constant	−78.61	−173.30 *	85.34	−141.04
	(57.81)	(94.88)	(241.50)	(100.46)
N	48	48	48	48
Adj R2	0.7738	0.8082	0.7084	0.7900

Note: Standard errors in paratheses, * *p* < 0.10 ** *p* < 0.05 *** *p* < 0.01.

## Data Availability

Data available from authors upon request.
